# Mechanisms of pelvic floor muscle training for managing urinary incontinence in women: a scoping review

**DOI:** 10.1186/s12905-022-01742-w

**Published:** 2022-05-13

**Authors:** Ying Sheng, Janet S. Carpenter, James A. Ashton-Miller, Janis M. Miller

**Affiliations:** 1 Indiana University School of Nursing, 600 Barnhill Dr., Indianapolis, IN 46202 USA; 2 Department of Mechanical Engineering, 2350 Hayward, Ann Arbor, MI 48109 USA; 3 Institute of Gerontology, University of Michigan, 300 N Ingalls St, Ann Arbor, MI 48109 USA; 4 Department of Health Behavior and Biological Science, School of Nursing, 426 N Ingalls St, Ann Arbor, MI 48104 USA; 5 Department of Obstetrics and Gynecology, Medical School, University of Michigan, 1500 E Medical Center Dr, Ann Arbor, MI 48109 USA

**Keywords:** Urinary incontinence, Kegel, Knack skill, Pelvic muscle exercises, Physical therapy, Transversus abdominis

## Abstract

**Background:**

Pelvic floor muscle training is recommended as first line treatment for urinary incontinence in women based on three proposed theorized mechanisms: ‘Enhanced Pelvic Floor Muscle Strength,’ ‘Maximized Awareness of Timing,’ and ‘Strengthened Core Muscles’. The purpose of this scoping review was to systematically map evidence for and against theorized mechanisms through which pelvic floor muscle training interventions work to reduce urinary incontinence in women.

**Methods:**

The scoping review is based upon a comprehensive search of relevant literature published from 1990 to 2020 in PubMed, CINAHL, PsycINFO, ClinialTrials.gov, reference lists from review articles, and hand searches of articles by known researchers in the field. We included English-language, peer-reviewed articles on pelvic floor muscle training as an intervention for adult women if they provided empirical evidence to testing the theorized intervention mechanisms. Two independent reviewers screened articles for inclusion and extracted data to describe details of each study (author, year, country, design, sampling), measures of pelvic floor muscle strength and urinary incontinence, statistical analysis of linkage between changes in the measures, and pelvic floor muscle training regimens. Data were summarized to facilitate the integration of diverse evidence to draw conclusions on supporting or refuting the three proposed theorized mechanisms for managing urinary incontinence in women.

**Results:**

Of the 278 articles identified with the search, 13 (4.7%) met inclusion criteria. There was weak to no evidence for the mechanism of enhanced pelvic floor muscle strength, equivocal support for maximized awareness of timing, and no evidence for strengthened core muscles.

**Conclusions:**

This review revealed extremely limited data supporting the proposed theorized mechanisms underlying pelvic floor muscle training programs to manage urinary incontinence in women. Such evidence is needed to help women and clinicians understand how, why and when a woman benefits from pelvic floor muscle training. Future studies should specifically state and report statistical analysis that relates the theorized mechanisms to the training outcomes observed.

**Supplementary Information:**

The online version contains supplementary material available at 10.1186/s12905-022-01742-w.

## Background

Urinary incontinence (UI) is defined as the report of any involuntary leakage of urine [1]. The global prevalence of UI is between 5 to 69% during a woman’s lifetime, with higher prevalence in older age groups [2, 3]. Pelvic floor muscle training (PFMT) is recommended as the first line treatment for the most common forms of incontinence: stress, urge, or mixed urinary incontinence (SUI, UUI, or MUI) [4]. PFMT programs vary but can be prescribed as taught and supervised by a health professional, conducted for the purpose of preventing or treating UI and other pelvic floor disorders [5]. Generally, referrals to PFMT programs (typically as supervised by physical therapists or physiotherapists), are routinely made by obstetricians, midwives, gynecologists, urologists, urogynecologists, nurse practitioner continence specialists, in addition to general health care providers.

**Fig. 1:**
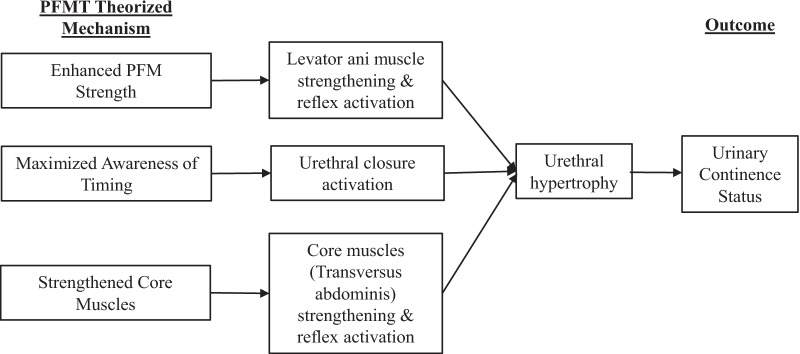
Theorized Mechanisms of PFMT. PFMT, pelvic floor muscle training; PFM, pelvic floor muscle

Three proposed theorized mechanisms guide current PFMT approaches [6] (Fig. 1). We named the theorized mechanisms according to the focus and goal of each. The first and dominant mechanism is “Enhanced Pelvic Floor Muscle Strength,” pertaining to increasing the cross-sectional area of the key support muscle underlying the urethra. This mechanism targets the levator ani muscle and is operationalized as repetitive contractions to exercise the levator ani muscle. The names of the PFMT programs that use this theorized mechanism are (most commonly) “Kegel’s exercises” or pelvic floor muscle (PFM) exercises.

The *second* mechanism is “Maximized Awareness of Timing” pertaining to enhanced personal control over improving urethral closure pressure momentarily in a moment of need [7]. This mechanism targets the urethral striated muscle, is operationalized as first identifying triggers for leakage so that anticipatory timing of PFM contraction in that moment can occur and as a PFMT program it has been named ‘the Knack’, ‘stress strategy’, and ‘perineal lock’. A timely contraction as an overarching descriptor for training emphasizes the knack of knowing when to contract across everyday life events.

The *third* mechanism is “Strengthened Core Muscles.” This mechanism is derived from the supposition that contraction of core abdominal muscles elicits a co-contraction of the PFM reflexively [8]. ‘Strengthened Core Muscles’ targets the transversus abdominis (TrA) muscle, and PFMT programs reliant on this theorized mechanism are typically referred to as core muscle training.

It is unclear how much evidence is available to support these theorized mechanisms. Reductions in UI in at least some study participants [4, 9] are a desired and logical outcome of these interventions but do not provide direct evidence of mechanisms. If data on mechanisms were available, it could be used to refine interventions and explain why some women do not benefit from certain treatments. For example, suppose there is empirical evidence showing that TrA strength increases after intervention and is highly associated with reductions in UI. Women who do not show increases in TrA strength would be expected not to show improved UI.

Reasons why a mismatch to theory occurred could be further investigated to refine the intervention. A mismatch to theory may be applicable only to certain subpopulations. For instance, it is now known that a partial or complete tear of critical PFM fibers away from their attachment at the pelvic bone occurs under certain childbirth-related conditions [10, 11]. The condition is chronic [12]. As a biological variable that directly affects ability to strengthen or even activate the PFM, a muscle detachment tear represents a logical moderating variable on hypothesized relationships between either maximizing strength or maximizing awareness as mechanisms underlying PFMT success. In this example, the likely assumption exposed is that all women have the biological capacity to contract the PFM, when in reality there is a categorical variable of PFM tear that logically interferes. Evidence for or against the mechanism is thus muddied if the moderator is not included.

Therefore to demonstrate these gaps, a scoping review of the literature was conducted to summarize evidence on the theorized mechanisms underlying PFMT for UI in women. Mechanisms that were investigated included enhanced PFM strength, maximized awareness of timing, and strengthened core muscles. The following research questions were formulated: What is known from statistical analyses in the literature about associations between changes in PFM strength and UI? What is known about PFM tear as a moderator of these associations?

## Methods

### Protocol and registration

This review of the literature was performed following the Preferred Reporting Items for Systematic reviews and Meta-Analyses extension for Scoping Reviews (Additional file 1: PRISMA-ScR) guidelines [13]. This review was not registered. Institutional Review Board approval was not applicable.

### Information sources and searches

We performed separate literature searches for each theorized mechanism because each required specific search terms. We searched PubMed for evidence pertaining to all three mechanisms. For enhanced PFM strength mechanism, as the earliest and most long-standing theorized mechanism, we added a search of PsycINFO and CINAHL as the historically gold standard databases for psychological, nursing, and behavioral approaches. For these search engines, search limiters were set to be adults age 18 or older, female, and articles in English published 1990 and after. The reason of choosing publications from 1990 was Wells (1990) conducted a similar state of the literature as reviewed, revealing 22 articles dating from 1952 to 1988 [14].

We then used hand search strategies to screen articles cited by systematic reviews of interventions, such as Cochrane reviews, and seminal PFMT articles written by researchers well-known in the field. We also searched ClinicalTrials.gov to find additional clinical trials that applied PFMT interventions to treat UI in women and then searched MEDLINE/PubMed using the National Clinical Trial number to identify the publications. The final search results were exported into EndNote, and duplicates were removed by the first author.

#### Enhanced PFM strength search details

The search for the enhanced PFM strength mechanism was completed in July 2020 excepting for the search in ClinicalTrials.gov, which was completed in December 2020. The literature was searched using various combinations of both Medical Subject Headings and/or related keywords. Specific search strings were: (pelvic floor muscle strength OR levator ani muscle OR Kegel muscle OR pelvic floor muscles) AND (pelvic floor muscle training OR Kegel exercises OR pelvic muscle exercises) AND urinary incontinence. The search strategy for PubMed using the above search strings as well as details of clinical trials search from ClinicalTrials.gov can be found in Additional file 2: Supplementary A.

#### Maximized awareness of timing search details

The search for the maximized awareness of timing mechanism was completed in PubMed in August 2020, excepting the ClinicalTrials.gov search completed in December 2020. This search included maximized awareness as either the singularly focused mechanism of PFMT or as a mechanism in addition to enhanced PFM strength as another proposed theorized mechanism and accompanying part in operationalizing the full PFMT reported. Search strings were stress urinary incontinence AND (knack OR stress strategy OR perineal lock OR single Kegel OR precontraction OR perineal co-contraction OR voluntary pelvic muscle contraction OR pelvic clutch OR bracing OR counter bracing OR quick Kegel OR perineal blockage).

#### Strengthened core muscles search details

The search for the strengthened core muscles mechanism was completed in PubMed in August 2020 and ClincialTrials.gov in December 2020. The search strings were (transverse abdominis training OR transverse abdominis exercises OR transversus abdominis training OR transversus abdominis exercises OR transversus abdominis muscle contraction) AND urinary incontinence.

### Eligibility criteria

Inclusion criteria common across the three searches were: peer-reviewed, original research, full-length articles; English-language; published in 1990 and later; PFMT as an intervention for adult women; and reported data on purported mechanisms. For the latter, we included articles that reported data on associations between increased PFM strength and incontinence reduction for the enhanced PFM strength mechanism; mechanism data of PFM volitional activation, urethral closure and striated muscle bulk for maximized awareness of timing mechanism; and mechanism data of co-contraction of PFM and TrA muscles as indicative of strengthened core muscles mechanism.

Common exclusion criteria were: book chapters, review articles, commentaries, dissertations, published abstracts, newspapers, magazines, animal studies, and studies or men or children (under 18 years of age).

### Selection of sources of evidence

Lists of potential articles were combined and de-duplicated. In the first stage of review, the first author screened abstracts and titles and retained only those that met inclusion criteria. In the second stage of review, the first author reviewed full texts and retained only those that met inclusion criteria. Disagreements were rare and were resolved through discussion.

We included all articles that met inclusion criteria. Nearly all included articles did not have a stated purpose of testing the mechanism of action of the intervention. Thus, it seemed inappropriate to employ traditional approaches to complete risk of bias in ratings. Rigorously performed trials specific to their specified trial outcomes for the intervention would have rated poorly when considering the purpose of this review—linking analysis of purported mechanism to intervention outcomes.

### Data charting process

The first and second authors independently reviewed full texts. The first author extracted relevant data from each eligible article and the second author verified the data extraction. Authors completed all data extraction using Microsoft Excel spreadsheets. Each column was a piece of data to be extracted and each row listed an article. The data extraction form was designed by the first author and revised as needed during the data extraction process.

### Data items

Data extracted from the studies included: authors, year of publication, country, statement regarding how sample size was established, sample characteristics, measures used for quantifying the purported theorized mechanism, measures used for quantifying incontinence, description of the PFMT regimen used, and PFM tears. We tabulated statistical parameters reflecting the linkage between purported mechanisms and intervention outcomes, such as correlation, odds ratios, and regression coefficients.

Heterogeneity of PFMT regimens, measurement instruments, and mechanism reports prohibited meta-analysis; thus, a scoping review was performed. We grouped the articles by the three individual theorized mechanisms, summarized sample characteristics, statistical analysis methods, the associations between changes in PFM strength and UI and interventions success.

Because our research questions emphasized robust testing of theorized mechanisms as described in the selected articles, data were also extracted on measures of PFM strength. Extraction categories were (a) the measure’s common or generic name, (b) type (subjective, objective), (c) purpose, and (d) psychometrics.

## Results

**Fig. 2:**
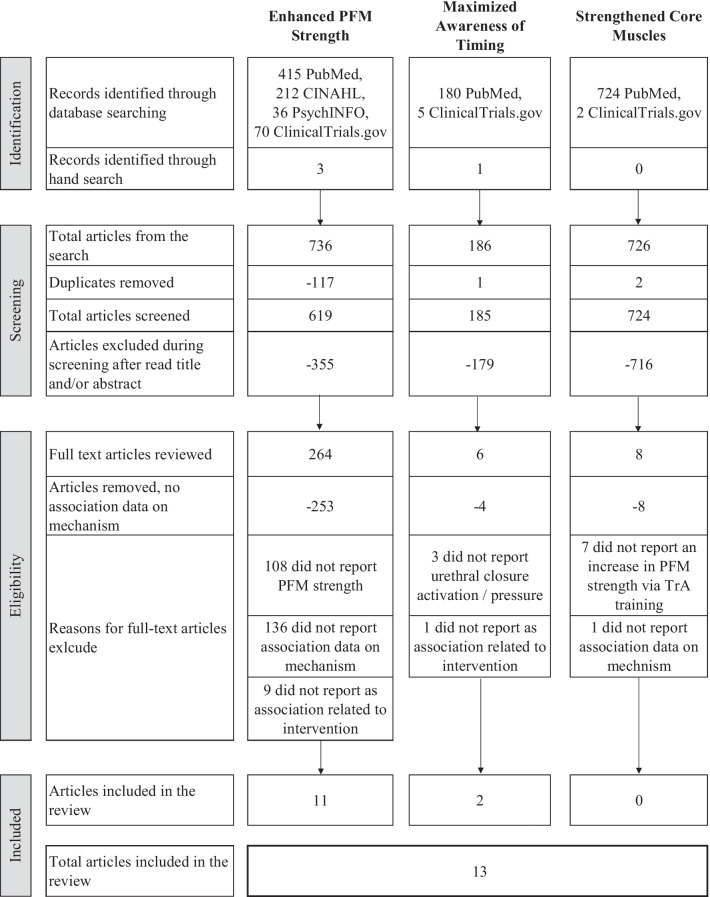
Modified PRISMA flow diagram showing disposition of articles from the three searches. PRISMA, Preferred Reporting Items for Systematic reviews and Meta-Analysis; PFM, pelvic floor muscle; TrA, transversus abdominis

Figure 2 shows the flow of articles from the three searches (one for each purported mechanism). Of 278 full articles reviewed, only 13 (4.7%) were identified as meeting inclusion criteria for providing any statistical analysis testing the linkage between purported mechanism and intervention outcomes. The results by each of the three mechanisms are described below.

### Mechanism 1. Enhanced PFM strength

As shown in Table 1, the 11 retained articles [15–25] were conducted in Norway, the USA, Turkey, Sweden, Taiwan, Japan, China, and Belgium and included a wide range of populations. Sample sizes assigned to PFMT ranged from 35 to 215. Four studies included control groups—three were no UI treatments and one was bladder training. Mean ages ranged from 26 to 77 years. Samples included pregnant women during the 20th to 34th week of pregnancy [16], postpartum women [24], middle aged and elderly parous women [17, 25], and women older than 70 years [19]. Most samples were characterized with SUI or SUI predominant if representing MUI. Studies included both subjective and objective measures of PFM strength and UI. All studies reported group level increases in PFM strength following intervention, and all but one [15] reported group improvements in UI (Table 2).

**Table 1:** Description of study samples and urinary incontinence type

Author, year and country	Study design	Sample				UI type		
		n Assigned PFMT	Age M ± SD, (age range)	Condition	n, Comparators	Urge	Stress	Mixed
*Mechanism 1. Enhanced PFM strength*								
Bø (2003), Norway	Secondary analysis of RCT	52	45.5, (24–64)				X	
Burns et al. (1993), USA	RCT	83	63.0 ± 6.0, 63.0 ± 5.0, (≥ 50)		40 controls		X	X
Dinc et al. (2009), Turkey	RCT	35	26.05 ± 4.8, 27.7 ± 7.2	20th-34th week of pregnancy	33 controls		X	X
Dougherty et al. (1993), USA	Pre and post-intervention study	65	51.3 ± 10.6, (35–75)	Mid-age and elderly Parous			X	
Hahn et al. (1993), Sweden	Case-controlled study	170	51.3 ± 0.98, (27–84)		27 awaiting surgery		X	
Hung et al. (2012), Taiwan	Prospective cohort study	68	50.5 ± 6.0				X	X
Kim et al. (2007), Japan	RCT, crossover follow up trial	70 (35 in each group)	76.6 ± 5.0, 76.6 ± 3.8	Elderly			X	
Nystrom et al. (2018), Sweden	Pre and post-intervention study	61	44.7 ± 9.7, (27–72)				X	
Segal et al. (2016), USA	Prospective cohort study	215	54.4 ± 12.7	Mid-age and elderly parous			X	
Sun et al. (2018), China	Prospective cohort study	133	33.63 ± 3.98, 42.66 ± 11.35	Postpartum			X	
Theofrastous et al. (2002), USA	RCT	69	60.6 ± 10.0, (45–79)		68, bladder training		X	X
*Mechanism 2. Maximized awareness of timing*								
Cammu et al. (2000)^a^, Belgium	10-Year follow-up	45	61.0 ± 10.0				X	
Junginger et al. (2014), Germany	Prospective cohort study	55	Not reported			X	X	X

Blank cells = information was not appliable or not available. Articles retained from search results (n = 13 including 11 for enhanced PFM strength mechanism and 2 for maximized awareness of timing mechanism). This table focuses on tabulation in readiness of integration of information for critical analysis of the general quality of studies on PFMT to treat UI, while also testing for theorized mechanism/s of effect

UI, urinary incontinence; PFMT, pelvic floor muscle training; PFM, pelvic floor muscle; M, mean; SD, standard deviation; RCT, randomized controlled trial

^a^A 10-year PFMT follow up article was also found in the main PFMT strengthening theory literature search, but it was not included in the analysis of results for the main PFMT strengthening theory since it did not report PFM strength and had no statistical analysis of correlation between changes in PFM strength and incontinence

**Table 2:** Description of pelvic floor muscle strength and urinary incontinence measures used

Author and year	PFM strength			UI		
	Subjective	Objective	Direction of change over time	Subjective	Objective	Direction of change over time
*Mechanism 1. Enhanced PFM strength*						
Bø (2003)		Vaginal balloon catheter	↑	Leakage index	Pad test	Not reported
Burns et al. (1993)		EMG	↑^a^	24-h urinary diary		↓
Dinc et al. (2009)		Perineometer	↑	3-day urinary diary	1-h pad test	↓
Dougherty et al. (1993)		Intravaginal balloon device	↑	24-h urinary diary	24-h pad test	↓
Hahn et al. (1993)	Vaginal palpation		↑		Pad test	↓
Hung et al. (2012)	Modified Oxford scale		↑	Severity Index score		↓
Kim et al. (2007)		Dynamometer	↑	Modified ICIQ questions		↓
Nystrom et al. (2018)	A self-rated PFM strength question		↑	PGI-I		↓
Segal et al. (2016)		Vaginal EMG	↑	Self-reported UI improvement		↓
Sun et al. (2018)		Vaginal manometer	↑		1-h pad test	↓
Theofrastous et al. (2002)		Balloon devices	↑	Urinary diary	48-h pad test	↓
*Mechanism 2. Maximized awareness of timing*						
Cammu et al. (2000)^b^	Vaginal palpation^c^		↑ Timely precontraction skill	Self-assessment		↓
Junginger et al. (2014)	Vaginal palpation^d^	Perineal ultrasound^e^	↑ Timely precontraction skill	Posttreatment improvement scales, analog scales for satisfaction, frequency of precontraction		↓

Blank cells = Information was not appliable or not available. Articles retained from search results (n = 13 including 11 for enhanced PFM strength mechanism and 2 for maximized awareness of timing mechanism). This table focuses on tabulation in readiness of integration of information for critical analysis of the general quality of studies on PFMT to treat UI, while also testing for theorized mechanism/s of effect. All pad tests for UI were performed under provocative maneuver to ask participants to, such as cough, pick, set up and down, run or walk, jump, step, bounce, rise, lay down sit-ups, or stand

PFM, pelvic floor muscle; UI, urinary incontinence; PFMT, pelvic floor muscle training; EMG, electromyography; ICIQ, International Consultation on Incontinence Questionnaire; PGI-I, Patient Global Impression of Improvement Questionnaire

^a^Pelvic muscle electromyography scores measured as quick and sustained contractions only increased in PFMT + biofeedback group, not in the PFMT only group

^b^A 10-year PFMT follow up article was also found in the main PFMT strengthening theory literature search, but it was not included in the analysis of results for the main PFMT strengthening theory since it did not report PFM strength and had no statistical analysis of correlation between changes in PFM strength and incontinence

^c^Vaginal palpation to correct PFM contraction

^d^Vaginal palpation to evaluate pelvic floor dysfunction

^e^Perineal ultrasound to evaluate pelvic floor dysfunction and for bladder-neck effective PFM contraction

The majority of the 11 studies provided *indirect* evidence of the association between improved PFM strength and UI (Table 3). Only two studies provided direct evidence of the correlation as part of the primary aims [15, 21], all others provided evidence as supplementary information.

**Table 3:** Statistical analysis of linkage between changes in pelvic floor muscle strength and urinary incontinence

Author and year	Evidence		Statistical tests of associations between changes in PFM strength and UI with treatment	Association findings by type of measures			
	Direct	Indirect		Subjective PFM strength and UI	Objective PFM strength, subjective UI	Subjective PFM strength, objective UI	Objective PFM strength and UI
*Mechanism 1. Enhanced PFM strength*							
Bø (2003)	X		0.23 (*p* = 0.05), 0.34 (*p* < 0.01) (Spearman’s rho)		Moderate		Weak
Burns et al. (1993)		X	0.26 (*p* < 0.005), 0.22 (*p* < 0.03) (Pearson’s r)		Weak		
Dinc et al. (2009)		X	− 0.17 (*p* = 0.34) and 0.06 (*p* = 0.75) (Pearson’s r)		NS		NS
Dougherty et al. (1993)		X	No value		NS		NS
Hahn et al. (1993)		X	No value (Pitman’s permutation test, *p* < 0.05)			Value not reported	
Hung et al. (2012)		X	0.265 (Spearman’s rho, *p* = 0.043); 0.238 (standardized coefficients, *p* = 0.014)	Weak			Weak
Kim et al. (2007)		X	No value; 4.545 (*p* = 0.10), 3.100^a^ (*p* = 0.21) (Cochran Q)		NS		
Nystrom et al. (2018)		X	35.54 (4.96–254.61) (OR, 95% CI; *p* < .0.001)	Moderate to large			
Segal et al. (2016)		X	No value (Spearman’s rho)		NS		
Sun et al. (2018)		X	1.042 (1.010–1.070) (OR, 95% CI)				Weak
Theofrastous et al. (2002)	X		0.32 (Pearson’s r, *p* = 0.04), other NS (*p* > 0.30)^b^		Moderate to NS		
*Mechanism 2. Maximized awareness of timing*							
Cammu et al. (2000)^c^		X	NS^d^				NS
Junginger et al. (2014)		X	− 0.36 (Spearman’s rho, *p* = 0.006)); 71% women routinely used Knack had less UI (*p* = 0.021)^e^	Moderate			

Blank cells = information was not appliable or not available. Only measures and changes that related to statistical analysis of correlation between the changes in PFM strength and incontinence were included in the table

PFM, pelvic floor muscle; UI, urinary incontinence; OR, odds ratio; NS, non significant; CI, confidence interval

^a^At 3 months for treatment group and at 12 months for both treatment and control groups

^b^Non significant correlations were found between increased PFM strength with reduction in incontinence episode per week and in pad weight, significant correlation = 0.32 for the correlation between an increase in maximum sustained vaginal pressure and reduction in incontinence episodes per week in women with stress incontinence

^c^A 10-year PFMT follow up article was also found in the main PFMT strengthening theory literature search, but it was not included in the analysis of results for the main PFMT strengthening theory since it did not report PFM strength and had no statistical analysis of correlation between changes in PFM strength and incontinence

^d^NS due to few participants—more often use Knack, the greater improvement; 3 leaked urine during stress test

^e^71% women routinely used Knack had less UI; improvement of symptoms was not associated with length of follow up and did not decrease over time

In a majority of studies, analysis of the relationship was based on correlation coefficients including Spearman’s rho, Pitman’s permutation test, or Pearson’s r. Few used logistic regression analysis (odds ratios [OR]), Cochran’s Q, or multiple linear regression analysis (standardized coefficients).

Most articles (n = 7 of 11) reported a statistically significant association of PFM strength change and incontinence change. Only one article [20] reported a moderate to large statistical association between self-reported improvement of PFM strength and PFMT success (defined as improved continence) but the confidence interval was exceptionally large (OR = 35.54, 95% CI 4.96–254.61). All others reported no or only weak or moderate associations.

Of the two articles that stated a primary aim of looking for correlation between purported mechanism and outcome, one reported moderate-to-weak and one reported no significant correlation. One article reported that improvement in maximal PFM strength measured by a vaginal balloon catheter significantly correlated with improvement in urine leakage index (rho = 0.34, *p* < 0.01) and with UI reduction in pad weights (rho = 0.23, *p* = 0.05) [15].

The second article reported inconsistent findings of the associations. Although the authors found a moderate correlation (r = 0.32, *p* = 0.04) between increased maximum sustained vaginal pressure measured via a balloon device and self-reported reduction in weekly incontinence episodes, they did not find significant correlations between increased PFM strength measured by the same balloon device with the self-reported incontinence reduction weekly episodes, or reduction of leakage amount in pad weights [21].

The remaining articles had no stated aim about linking mechanism and outcome. Three studies reported weak correlations between purported mechanism and UI reduction (rho = 0.265, *p* = 0.043; r = 0.22, *p* < 0.03 and r = 0.26, *p* < 0.005; or OR = 1.042, 95% CI 1.010–1.070, respectively) [18, 22, 24]. One article also reported standardized coefficients (= 0.238, *p* = 0.014) for increased PFM strength correlated with reduction in UI severity index in a multiple regression analysis, controlling for baseline levels [18].

One article did not report the parameter value of a correlation reported as significant [23]. Another four studies did not find any significant correlation between change in PFM strength and change in incontinence [16, 17, 19, 25], with one [19] reporting non-significant Cochran Q-values = 4.545 (*p* = 0.10) at 3 months for intervention group and 3.100 (*p* = 0.21) at 12 months follow up for all women after received PFMT treatment between improved (adductor muscle) strength and proportion of cured women.

Details of the PFM strength measures used in these studies and their limitations are described in Table 4 and Additional file 3: Supplementary B. Heterogeneity in instruments and accompaniment measurement limitations are notable, making it impossible to compare results across articles. Similarly, there is enormous variance in exercise regimens of the “Kegel muscle”, as shown in Table 5 and Additional file 3: Supplementary B, even though all purportedly are operating from the shared theoretical mechanism to enhance strength of the muscle supporting the urethra.

**Table 4:** Instruments used in the reviewed studies to measure pelvic floor muscle strength

	Measures common name (review article)	What is assessed	Reliability	Validity
Subjective	Vaginal digital examination [23, 26]	Grading *contraction, pressure* around finger/s (4-point scale)	–	–
	Modified Oxford scale [18]	Grading *contraction, pressure* around fingers, Poisson effect (as muscle is contracted, it expands a bulging up) (6-point scale)	r = 0.27–0.95 (inter-rater) [49–53] r = 0.93 (test–retest) [50]	Correlation with perineometric pressure 0.79 [50] Contaminated by IAP
	A self-rated PFM strength question [20]	Self-reported improvement of PFM strength	–	–
Objective	Perineometer-like devices [16, 24]	Maximum voluntary vaginal closure *pressure* (mm Hg)	r = 0.79–0.80 (inter-rater) [54]	Good agreement with Brink digital exam score [55] Contaminated by IAP
	Intravaginal balloon-like device [15, 17, 21]	Maximum voluntary vaginal closure *pressure* (mm Hg)	r = 0.52–0.85 (test–retest) [56]	Contaminated by IAP [57]
	Needle EMG: quantitative EMG [22]	Muscle *electrical activity* from individual motor units (in microvolt units)	r = 0.89 (range 0.78–0.95) [22] hard to repeat	Not possible to measure contractile force using EMG [58] Contaminated by IAP
	Surface EMG (vaginally): quantitative EMG [25]	Muscle *electrical activities* from summated from many motor units (millivolt)	Between-visit ICC ranging 0.76–0.97 [59]	Measured PFM activity other than vaginal closure pressure Contaminated by adjacent muscles [60, 61]
	A handheld dynamometer (mTasMF-01, ANIMA, Japan) [19]	Hip adductor muscle strength	–	Not measure PFM strength
	Sagittal dynamic (perineal) ultrasound [27]	Cephalic displacement (in mm) of the bladder neck in a sagittal view available as biofeedback (as opposed to caudal movement observable when she pushes down instead)	r = 0.52–0.96 (intra-rater) [62]	Visual “lift” of the bladder neck with a correct PFM contraction

—, Information was not available; IAP, intraabdominal pressure; PFM, pelvic floor muscle; EMG, electromyography; ICC, intra-class correlation coefficients

**Table 5:** Details of pelvic floor muscle training regimens

Author and year	PFMT at home dose			PFMT in clinic dose			Graded training	Duration	PFMT delivery					Methods for compliance
	# Sessions/day	# Contractions/session	Minutes/session	# Sessions/week	# Contractions/session	Minutes/session			Individual	Group	Supervised	Unsupervised	Device assisted training	
*Mechanism 1. Enhanced PFM strength*														
Bø (2003)	3	3 sets of 8–12 per day^a^		1				6 mo	X	X	X	X		Meet wkly for intensive group
Burns et al. (1993)		4 sets of 20 (10 quick + 10 sustained), then ↑ 10 per set to 200		1		25–35	X	8 wk^b^	X		X	X	Biofeedback (1 of 2 groups)	Call and exercise reminder cards
Dinc et al. (2009)	2–3	3 sets of 10–15					X	12–28 wk	X			X		
Dougherty et al. (1993)	3/wk	15–45					X	16 wk	X			X	Audio recording	Call and written records wkly
Hahn et al. (1993)	6–8	Maximal, during jumping and coughing		1 × 4–5 wks then monthly for measures				1–18 mo (mean 4.7 ± 0.2)	X			X		
Hung et al. (2012)	≥ 3	6 high-intensity (hold 10 s and rest 10 s)						4 mo	X			X		
Kim et al. (2007)	≥ 2 for follow up	2–3 sets	30 for follow up	2 then monthly^c^	20^a^	60		12 wk^d^	X	X	X	X	Fitness exercises	
Nystrom et al. (2018)	3	6 basic + 6 advance					X	3 mo	X			X	App-based + Knack	
Segal et al. (2016)	2		1–7	1/two wks	3		X	16 wk	X		X		FemiScan trainer device	
Sun et al. (2018)	3	10 slow + 3–4 fast^a^	20	2		20		6–8 wk	X		X	X	Biofeedback + electrical stimulation^e^	
Theofrastous et al. (2002)	2	5 quick + 10–20 sustained		1 × 4 wks		30	X	12 wk	X		X	X	Biofeedback	
*Mechanism 2. Maximized awareness of timing*														
Cammu et al. (2000)^f^		As frequently as possible + Knack		2	20	30	X	10 wk	X		X	X		
Junginger et al. (2014)		Engage precontraction into daily life		Median 2 (1–6) for study period	?^g^	15–90 (total 60–240)		4–6 wk^h^	X		X	X	Perineal ultrasound	

Blank cells = information was not appliable or not available. Articles retained from search results (n = 13 including 11 for enhanced PFM strength mechanism and 2 for maximized awareness of timing mechanism). This table focuses on tabulation in readiness of integration of information for critical analysis of the general quality of studies on PFMT to treat urinary incontinence, while also testing for theorized mechanism/s of effect

PFMT, pelvic floor muscle training; PFM, pelvic floor muscle; mo, month; wk, week; TrA, transversus abdominis

^a^Performed in supine, sitting, and standing positions with legs apart

^b^Follow up at 3 months and 6 months

^c^Added 2 times 60-min exercise sessions plus fitness exercises for 12 weeks and then monthly for 12 months

^d^Follow up at 12 months

^e^For women with two times incontinence after completed basic training or with week strength for active training

^f^A 10-year PFMT follow up article was also found in the main PFMT strengthening theory literature search, but it was not included in the analysis of results for the main PFMT strengthening theory since it did not report PFM strength and had no statistical analysis of correlation between changes in PFM strength and incontinence

^g^TrA precontraction + Knack + urge strategies, number of contractions was not clear from the article

^h^Follow up at 1 to 16 months (median 7, mean 7.6)

### Mechanism 2. Maximized awareness of timing

The two studies included in the review had small sample sizes and applied maximized awareness of timing as well as enhanced PFM strength in operationalizing the PFMT programs (Tables 1, 2). One [26] studied 45 women with SUI and used vaginal palpation to correct PFM contractions. Another one [27] studied 55 women who had MUI, SUI or overactive bladder and used both vaginal palpation and perineal ultrasound to evaluate pelvic floor dysfunction and perineal ultrasound was also used to ensure bladder neck effective PFM contractions. Neither reported PFM strength. Both studies described that women gained PFM skill in voluntarily contracting the PFM or improved PFM function and also found self-reported improvement of UI and success for some women.

Both articles provided indirect evidence for the theorized mechanism of maximized awareness of timing (Table 3) based on different measures (Tables 2, 4). In both articles [26, 27], frequency of application of maximized awareness was described as being related to success in terms of improved continence and treatment satisfaction. However, that relationship was not significant in one article [26] and only weakly moderate in another [27]. The latter found maximized awareness frequency of use was only minimally correlated with patient satisfaction with PFMT (rho = − 0.36, *p* = 0.006).

Both studies involved using the Knack in daily life to treat UI although the PFMT regimens were different. Details of the Knack combined with PFMT regimen used in the two studies are described in Table 5 and Additional file 3: Supplementary B.

### Mechanism 3. Strengthened core muscles

As shown in Fig. 2, all articles were excluded during full text review because they did not have analytic data linking mechanism and PFMT outcome. Thus, there were no articles reporting data on the purported mechanism of strengthened core muscle.

### PFM tear as a moderating variable

No studies evaluated PFM tears.

## Discussion

The overall findings from this review are concerning. Results show that the state of the science has a near complete lack of evidence to support or refute the three purported mechanisms through which PFMT interventions are believed to work (Fig. 1). Analytic parameters used to examine the association varied in the reviewed studies. Almost all of the studies reported no or weak associations.

Respectively, less than 5%, less than 1% and 0% of articles provided confirmation for enhanced PFM strength, maximized awareness of timing, or strengthened core muscles as the purported mechanism of PFMT interventions. Thus, the overall finding is that because theorized mechanisms are not properly investigated, these mechanisms are largely assumed to be operating, rather than empirically shown to be responsible for intervention effects. Assumptions about how interventions are efficacious/effective need further testing. Our findings are comparable to a similar state of the literature as reviewed by Wells (1990) revealing 22 articles dating from 1952 to 1988, and concluding “the research data is limited in … quality of design, and extent of reporting” [14].

Although the mechanism of urgency differs from SUI, findings from our review may raise awareness for how the treatments for urgency work. Urge suppression strategy (Quick squeezing) is considered effective to reduce the feeling of urgency by quick contracting PFM and relaxing the bladder (diminish bladder detrusor muscle activation). To study the association between changes in PFM strength or how fast the PFM will be activated and change of the occurrence rates of urgency might provide some information to discover the underlying mechanisms of using the quick squeezing strategy to diminish urgency. Additional moderating variables, such as women with conditions such as diabetes and age should be considered along with the moderating variable of a tear highlighted in this review, since all of these conditions may influence the effectiveness of the quick squeezing.

### Lack of empirical support for theorized mechanisms

Most studies were underpowered for the association analysis. The studies with larger study samples reported weak significant associations, while the largest [25] and smallest [16] samples did not find significant associations. Future studies need to be designed so they are appropriately powered to directly test the association between changes in factors considered as the mechanism of interest and change in UI outcomes within and across subgroups. Future studies should include reporting of not only the average effect of the treatment, but also responders to PFMT, and associations related to purported mechanisms while controlling factors, such as sample size and age.

A majority of the reviewed studies used correlation coefficients that estimate the monotonic association between the changes in PFM strength and UI. No studies evaluated variations in the strength of the association between strength/skill and UI according to age, clinical condition, etc.

We emphasize that evaluating potential moderating variables can help determine which individuals are best able to achieve the mechanistic goals. For example, since PFM function declines with age [28], the age of the sample could affect the association between strength or skill and changes in UI. Certainly striated muscle in older women can be strength trained [29], but these muscles may never reach the strength of younger women because of the age-related loss of large diameter motoneurons and their associated large diameter muscle fibers. Future studies should apply scatterplots to graph the association between changes of PFMT strength and UI and multiple analytic parameters such as correlation coefficients and multiple regression models controlling covariates to examine the association.

Study measures to test theorized mechanism were highly variable, subject to examiner influences, and of questionable and varying validity. These review findings are aligned with findings from another review [30] reporting lack of validity and reproducibility when using the Oxford Grading scale to measure PFM strength. For example, PFM strength measures were contaminated by intraabdominal pressure when measuring during effort at PFM contraction.

There is a lack of reliable measures of PFM strength at all within in-clinic practices. One previous review found that most PFMT studies failed to measure the treatment outcomes [14]. For instance, work on the issue of crosstalk from changes in intraabdominal pressure on measurements of PFM strength is decades long but while newer instruments have solved the problem through clever design, these improved instruments are not readily available in the clinic setting. This example, as well as many others that could be provided, highlight that without accurate, reproducible and reliable measures, lead one to the conclusion that it is presently simply not possible to evaluate the theorized underlying mechanisms.

PFMT regimens were highly variable, interfering with synthesis across studies. This heterogeneity makes it impossible to deduce which regimen(s) produce(s) the strongest evidence for a purported mechanism to treat UI. Regimens varied enormously with, astoundingly, no two studies from the literature reporting the same regimen despite commonality in underlying purported mechanism for building the PFMT program.

It has been previously reported that although almost all studies use either therapist supervision, intensive exercises, gradually increasing training load, or device-assisted training to ensure a woman contracts the correct muscles, almost all studies rate compliance to PFMT, but no one training regimen stands out from all the rest in terms of efficacy [31]. Three studies [18, 20, 21] had PFMT regimens with exercise at least three times per day with a minimum of two non-consecutive days per week, with one set of 8 to 12 maximum contractions reported significant associations. These PFMT regimens are consistent with the resistance training recommended by the American College of Sports Medicine [32]. Further studies should evaluate the theorized mechanisms of PFMT interventions and may design their PFMT interventions following the recommendation.

### Skill of maximizing awareness of timing developed during PFM strength training

Despite weak correlation or no correlation between changes in strength and incontinence, continence status improved. What could the underlying mechanism be for this effect? One might speculate that if a woman can maintain a certain degree of urethral closure pressure; she may be able to maintain continence if not too physically active. Thus, increasing PFM strength may not be necessary if women already have adequate PFM strength (the degree has not been identified to date). Being able to reliably recruit the urethral closure muscles to increase urethral closure pressure during those times of need when a woman anticipates urine leakage may suffice to manage leakage. Unfortunately, studies did not consider or evaluate this or any other alternative mechanisms.

Though PFM strengthening per se is not a goal of PFMT for timely contraction, it is possible that by learning the skill and habit of consciously activating the PFM at a moment of risk for leakage, the muscle over time may gain strength related to the new habit of timely contraction.

Voluntary PFM contraction during coughing can be trained and the reaction time of the contraction was improved via a 12-week PFMT program [33] or a 2-week cognitive rehabilitation (dual-task method) [34]. This might have led to urethral striated muscle hypertrophy, but we found no study that tested for the association of maximized awareness of timely contraction skill and either increased PFM strength or increased volitional urethral closure pressure.

There is evidence that urethral closure pressure may be enhanced in the moment of effort to contract the PFM [7, 35], and that urethral sphincteric hypertrophy which occurred with PFM strength training may be bolstered by maximized awareness of timely contraction training [36]. An earlier study [37] also demonstrated that even with complete bilateral tears of the “Kegel” muscles, activation of the urethral striated muscle was demonstrable in most women, though with less of a urethral closure pressure rise compared to women with intact PFM muscles. Pragmatically, women’s ability to feel and volitionally contract the small urethral striated muscle in isolation of the larger PFM has not been well demonstrated.

### Potential impact of a PFM tear on theorized mechanisms

None of the articles evaluated birth-related nonrepairable tears of the critical urethral support provided by the pubovisceral portion of the levator ani muscle (also referred to as Kegel muscle, pubococcygeal muscles, generally PFM). Such tears were suspected as early as 1943 [38] and irrefutable evidence suggests they are permanent [12].

Suggested psychometrically sound methods to diagnose PFM tear include various clinical appraisal estimates using one or more fingers for palpatory assessment, MRI, and three- or four-dimensional (3D or 4D) ultrasound for imaging assessment [38–46]. Importantly, though PFMT targets this very muscle whether women had a tear was not assessed in any of the articles. For these women, PFMT may strengthen the urethral striated muscle as an alternative mechanism explaining its effects on UI.

More evidence is needed, particularly given that 5% to 15% of women who have delivered vaginally have at least a partial tear of the pubovisceral muscle origin at the pubic bone [12, 41, 47]. In addition, our previous study found that postpartum women with PFM tears were more likely to have lower urethral closure pressure during Kegel exercises [48]. More research is needed to account for the effect of PFM tear(s) on theorized mechanisms as well as its effects of PFMT on reducing UI.

### Strengths and limitations

There are both strengths and weaknesses to our approach. The weaknesses include the fact that the databases searched were selected based on best match per guidance from a professional librarian specializing in instruction; however, the search was not fully exhaustive. Restriction to the English language may have excluded studies that might otherwise have offered unique and important data. Similarly, the exclusion of the grey literature may have meant we missed some articles.

This study focused on examining the PFMT literature for its robustness regarding the theoretical mechanisms underlying formation of the programs. Our findings of major gaps should not be misinterpreted as denigrating PFMT for it is well established that PFMT, in its many forms, can work for many women. Rather, our results call for research that is dedicated to better understanding theorized mechanisms to improve efficacy—in other words, we need greater understanding of why PFMT works.

Our literature review differs from others in the field of PFMT in its organization and focus on theoretical mechanisms. Our authorship includes experts in both PFMT mechanisms and interventions for pelvic floor disorders. Questions raised by the findings and implications for additional research lead to the suggestion that future investigators (1) report the theory base of their specific PFMT program, and (2) measure the constructs implied by the theory (e.g., PFM strength change) with psychometrically sound instruments.

Once this work is accomplished, cost-effective and time efficient treatment can become more of the norm. It may be time for an adaptive or even smart trial design to be used to identify how best to personalize PFMT in order to optimize a women’s outcomes by applying the theory-driven PFMT program most appropriate to her unique situation.

## Conclusions

Explicit theories for guiding PFMT research and practice are available in the literature, but statistical analyses for testing these purported theoretical mechanistic links with PFMT outcomes, to explain why some women respond and others do not, are largely absent. Future studies should explicitly state the theoretical basis guiding the work, which components of the theory are being tested, and the statistical analysis used to confirm the mechanism underlying the intervention-outcomes relationship, with attention paid to important potentially moderating variables, such as whether the levator ani is intact, age, and other important demographics. Only then can a better understanding of the mechanisms underlying PFMT for improving continence status be obtained.

## Supplementary Information

**Additional file 1.** Preferred Reporting Items for Systematic reviews and Meta-Analyses extension for Scoping Reviews checklist.

**Additional file 2.** Search strategies in PubMed and ClinicalTrials.gov for enhanced pelvic floor muscle strength mechanism.

**Additional file 3.**Measurement issues and pelvic floor muscle training regimens.

## Data Availability

The datasets used and/or analyzed during the current study are available from the corresponding authors on reasonable request.

## Abbreviations

UI: Urinary incontinence
PFMT: Pelvic floor muscle training
SUI: Stress urinary incontinence
UUI: Urge urinary incontinence
MUI: Mixed urinary incontinence
PFM: Pelvic floor muscles
TrA: Transversus abdominis
PRISMA-ScR: Preferred Reporting Items for Systematic reviews and Meta-Analyses extension for Scoping Reviews
CINAHL: Cumulative Index to Nursing and Allied Health Literature
