# Supplementary material for: Mechanisms of pelvic floor muscle training for managing urinary incontinence in women: a scoping review
Source: BMC Womens Health. 2022 May 13;22:161. doi: 10.1186/s12905-022-01742-w (PMC9103460; doi:10.1186/s12905-022-01742-w)
Supplement: Supplementary file 2 — Additional file 2. Search strategies in PubMed and ClinicalTrials.gov for enhanced pelvic floor muscle strength mechanism. [file 12905_2022_1742_MOESM2_ESM.docx]

**Supplementary A** provides the full search strategies in PubMed and ClinicalTrials.gov for Enhanced Pelvic Floor Muscle Strength using specific search strings and clinical trial numbers.

**1.**

PubMed search for Enhanced Pelvic Floor Muscle Strength was performed on August 31, 2020.

Search ((((((pelvic floor muscle strength[MeSH Terms]) OR levator ani muscle[MeSH Terms]) OR kegel muscle[MeSH Terms]) OR pelvic floor muscles[MeSH Terms])) AND (((pelvic floor muscle training[MeSH Terms]) OR kegel exercises[MeSH Terms]) OR pelvic muscle exercises[MeSH Terms])) AND urinary incontinence[MeSH Terms] Filters: English; Female; 80 and over: 80+ years; Aged: 65+ years; Middle Aged: 45-64 years; Middle Aged + Aged: 45+ years; Adult: 19-44 years; Young Adult: 19-24 years; Adult: 19+ years; Publication date from 1990/01/01

**2.**

Clinical trials search for Enhanced Pelvic Floor Muscle Strength was completed on December 15, 2020.

Consulted with an experienced librarian from the university medical library, the first author searched for clinical trials from ClinicalTrials.gov. Clinical Trial numbers were found from ClinicalTrials.gov listed as ClinicalTrials.gov Identifier.

Search in ClinicalTrials.gov, resulting in 77 trials (National Clinical Numbers). The librarian confirmed the same results.

SEARCH QUERY:

Condition or disease: urinary incontinence

Other term: “pelvic floor muscle strength” OR “levator ani muscle” OR “kegel muscle” OR “pelvic floor muscles”

Study type: All studies

Status: Recruitment (checked completed and unknown status)

Study results: All studies

Study results: All studies

age: Adult (18–64)

Older Adult (65+)

Sex: Studies with female participants

Intervention/treatment:  “pelvic floor muscle training” OR “kegel exercises” OR “pelvic muscle exercises”

Study start: from 01/01/1990

The first author checked the publications referenced in the trial records and found 33 relevant articles.

The first author also manually searched MEDLINE/PubMed for all National Clinical Numbers using the “Secondary Source ID” field and the OR operators between each NCT Number to find the relevant articles, filtered to English, Female, and Adult. 56 Clinical Numbers were not found in PubMed (possible reasons: NLM began to include the Clinical Trials registry number in the MEDLINE record when the number was published as part of an original paper. The numbers were not found in PubMed, which means no published articles were produced from those particular trials, or the authors did not include the NCT number in the resulting publication(s)).

**((((((((((((((((((((((((((((((((((((((((((((((((((((((((((((((((((((((((((((NCT04140253[Secondary Source ID]) OR (NCT02924740[Secondary Source ID])) OR (NCT01940432[Secondary Source ID])) OR (NCT01245153[Secondary Source ID])) OR (NCT02275728[Secondary Source ID])) OR (NCT02664714[Secondary Source ID])) OR (NCT03875196[Secondary Source ID])) OR (NCT03514147[Secondary Source ID])) OR (NCT01499784[Secondary Source ID])) OR (NCT04577872[Secondary Source ID])) OR (NCT03058042[Secondary Source ID])) OR (NCT02427230[Secondary Source ID])) OR (NCT03401983[Secondary Source ID])) OR (NCT01811602[Secondary Source ID])) OR (NCT03632447[Secondary Source ID])) OR (NCT02616952[Secondary Source ID])) OR (NCT02676700[Secondary Source ID])) OR (NCT01069484[Secondary Source ID])) OR (NCT02614105[Secondary Source ID])) OR (NCT03042351[Secondary Source ID])) OR (NCT04525846[Secondary Source ID])) OR (NCT01578369[Secondary Source ID])) OR (NCT03500185[Secondary Source ID])) OR (NCT00222248[Secondary Source ID])) OR (NCT04339010[Secondary Source ID])) OR (NCT03349736[Secondary Source ID])) OR (NCT00177541[Secondary Source ID])) OR (NCT03203798[Secondary Source ID])) OR (NCT01806350[Secondary Source ID])) OR (NCT00498888[Secondary Source ID])) OR (NCT00910338[Secondary Source ID])) OR (NCT00270738[Secondary Source ID])) OR (NCT02617472[Secondary Source ID])) OR (NCT01763762[Secondary Source ID])) OR (NCT00190606[Secondary Source ID])) OR (NCT00740428[Secondary Source ID])) OR (NCT04584359[Secondary Source ID])) OR (NCT02549729[Secondary Source ID])) OR (NCT02319096[Secondary Source ID])) OR (NCT02510131[Secondary Source ID])) OR (NCT02851719[Secondary Source ID])) OR (NCT00551551[Secondary Source ID])) OR (NCT04253925[Secondary Source ID])) OR (NCT01948713[Secondary Source ID])) OR (NCT04036604[Secondary Source ID])) OR (NCT03000647[Secondary Source ID])) OR (NCT00763984[Secondary Source ID])) OR (NCT03727269[Secondary Source ID])) OR (NCT01032265[Secondary Source ID])) OR (NCT02047032[Secondary Source ID])) OR (NCT03296462[Secondary Source ID])) OR (NCT01073878[Secondary Source ID])) OR (NCT00223821[Secondary Source ID])) OR (NCT02423005[Secondary Source ID])) OR (NCT01602107[Secondary Source ID])) OR (NCT00270998[Secondary Source ID])) OR (NCT03213522[Secondary Source ID])) OR (NCT00197314[Secondary Source ID])) OR (NCT00506766[Secondary Source ID])) OR (NCT03097549[Secondary Source ID])) OR (NCT01445834[Secondary Source ID])) OR (NCT03536923[Secondary Source ID])) OR (NCT00506116[Secondary Source ID])) OR (NCT02452593[Secondary Source ID])) OR (NCT00970320[Secondary Source ID])) OR (NCT01599715[Secondary Source ID])) OR (NCT04484753[Secondary Source ID])) OR (NCT01520948[Secondary Source ID])) OR (NCT02206958[Secondary Source ID])) OR (NCT01848938[Secondary Source ID])) OR (NCT02039830[Secondary Source ID])) OR (NCT03862326[Secondary Source ID])) OR (NCT00090584[Secondary Source ID])) OR (NCT00164138[Secondary Source ID])) OR (NCT02617797[Secondary Source ID])) OR (NCT03520426[Secondary Source ID])) OR (NCT00597935[Secondary Source ID]) Filters: English, Female, Adult: 19+ years**

**The following terms were not found in PubMed**: NCT01245153, NCT02275728, NCT02664714, NCT03875196, NCT03514147, NCT01499784, NCT04577872, NCT03058042, NCT02427230, NCT03401983, NCT01811602, NCT03632447, NCT02616952, NCT02676700, NCT02614105, NCT03042351, NCT04525846, NCT03500185, NCT00222248, NCT04339010, NCT03349736, NCT03203798, NCT01806350, NCT00498888, NCT00910338, NCT02617472, NCT00190606, NCT04584359, NCT02319096, NCT02510131, NCT02851719, NCT04253925, NCT01948713, NCT04036604, NCT03727269, NCT03296462, NCT01073878, NCT00223821, NCT02423005, NCT03213522, NCT00197314, NCT00506766, NCT03097549, NCT01445834, NCT03536923, NCT00506116, NCT02452593, NCT00970320, NCT01599715, NCT04484753, NCT01520948, NCT02206958, NCT03862326, NCT00164138, NCT02617797, NCT03520426

The following National Clinical Numbers were found in PubMed, resulting in 37 articles related to these National Clinical Numbers.

"NCT04140253"[Secondary Source ID] OR "NCT02924740"[Secondary Source ID] OR "NCT01940432"[Secondary Source ID] OR "NCT01069484"[Secondary Source ID] OR "NCT01578369"[Secondary Source ID] OR "NCT00177541"[Secondary Source ID] OR "NCT00270738"[Secondary Source ID] OR "NCT01763762"[Secondary Source ID] OR "NCT00740428"[Secondary Source ID] OR "NCT02549729"[Secondary Source ID] OR "NCT00551551"[Secondary Source ID] OR "NCT03000647"[Secondary Source ID] OR "NCT00763984"[Secondary Source ID] OR "NCT01032265"[Secondary Source ID] OR "NCT02047032"[Secondary Source ID] OR "NCT01602107"[Secondary Source ID] OR "NCT00270998"[Secondary Source ID] OR "NCT01848938"[Secondary Source ID] OR "NCT02039830"[Secondary Source ID] OR "NCT00090584"[Secondary Source ID] OR "NCT00597935"[Secondary Source ID] AND ((female[Filter]) AND (english[Filter]) AND (alladult[Filter])) Filters: English, Female, Adult: 19+ years

A total of 70 articles related to the trials were found from publications referenced in the trial records (33 articles) in Clinicaltrials.gov and PubMed search the National Clinical Numbers using “Secondary Source ID” (37 articles).
