# Supplementary material for: Mechanisms of pelvic floor muscle training for managing urinary incontinence in women: a scoping review
Source: BMC Womens Health. 2022 May 13;22:161. doi: 10.1186/s12905-022-01742-w (PMC9103460; doi:10.1186/s12905-022-01742-w)
Supplement: Supplementary file 3 — Additional file 3.Measurement issues and pelvic floor muscle training regimens. [file 12905_2022_1742_MOESM3_ESM.docx]

**Supplementary B**

**Measurements of pelvic floor muscle strength and urinary incontinence**

There is concern that some instruments did not measure pelvic floor muscle (PFM) strength alone. For example, most measures do not differentiate between increases in intraabdominal pressure and increases due to PFM contraction [1]. There was large variability in measurement psychometrics with some values indicating poor reliability or poor precision. For example, PFM strength was measured by an intravaginal device or by fingers placed vaginally. The device (e.g., balloon devices, dynamometer, or perineometer) reports the signal occurring, whereas the examiner reports on the perceived sensation on the fingers (e.g., modified Oxford scale) when a woman is asked to perform a PFM contraction. Kappa for the modified Oxford scale ranged from 0.27 to 0.95 [2-5] and test-retest reliability for intravaginal balloon devices ranged from 0.52 to 0.85 [6].

**Kegel’s exercises regimen**

There was variability across Kegel’s exercise regimen dosing, delivery, support, and supports to increase compliance for strength training. Of the 11 articles, four focused on Kegel’s exercises at home only [7-10]; all others combined therapist supervision in individual or group exercises with home exercises. All home sessions were unsupervised, all office sessions were supervised. Dosing of at home Kegel’s varied widely in relation to number of sessions per week or day, length of sessions, and number of contractions per session. Dosing of office Kegel’s exercise sessions also varied widely in relation to the number of sessions per week or month, length of each session, and number of contractions.

Many studies did not provide details on session length, whether at home or in office, or number of contractions. Length of the study period also varied from six weeks to 18 months. The total numbers of contractions daily varied across the Kegel’s exercises regimens. In addition, some Kegel’s exercises programs asked women to perform the contractions in different positions such as sitting, standing, or supine [11, 12], with fast or slow contraction [11], sustained contraction [13, 14], or during jumping or coughing [15].

Support for performing pelvic floor muscle training (PFMT) (Kegel’s exercises) and increasing the treatment effect included therapist supervision, assistance of biofeedback [11, 13, 14], audio cassette tape recording [10], added fitness exercises [16], using an application and adding Knack timing [8], or trainer device [17] to increase awareness of the contractions, with one study [11] added biofeedback and electrical stimulation only for women who had two incidents of incontinence after completing basic training or had weak PFM strength from active training. The majority of these studies delivered Kegel’s exercise training in person, except one study [8] which used an application. Women were required to meet with a therapist weekly, or receive telephone calls and exercise reminder cards, or submit written records regularly per week during the study period to increase compliance of with the Kegel’s exercise program [10, 12, 14].

**The Knack combined with pelvic floor muscle training regimen**

Both studies involved Maximized Awareness of Timing in daily life although the PFMT regimens were different (Table 5). One [18] asked women to attend a 30-minute private office visit to perform 20 contractions (10 forceful + 10 slow maximal) under supervision while gradually increasing the number of contractions in later visits according to the women’s capacity, twice per week for 10 weeks. Women were also asked to practice the contractions and the skill of contracting to maximize urethral closure pressure at home as frequently as possible. Another one [19] applied a specific PFMT rehabilitation program that consisted of a bladder neck effective PFM contraction, precontraction before coughing (the Knack), precontraction before urge triggers (urge strategies), and a co-contraction of transversus abdominis muscle, with no formal PFMT purposely for strengthening. Women were encouraged to use the precontraction in daily life. Women with overactive bladder also received behavioral advice and were instructed to perform submaximal and gentle precontractions to delay urgency. Perineal ultrasound was used as biofeedback to assist contractions during office sessions. The initial session lasted about 60 minutes and additional sessions were added as needed (Md=2, range 1-6). Each session lasted between 15 and 90 minutes, with a total of treatment time of 60 to 240 minutes (Md=120 minutes, M=133 minutes) for 4 to 6 weeks. Follow up was at 1 to 16 months (Md=7, Mean=7.6). No statistics on the association between the transverse abdominus training component and UI were reported.

**References**

1. Ashton-Miller JA, Zielinski R, DeLancey JO, Miller JM. Validity and reliability of an instrumented speculum designed to minimize the effect of intra-abdominal pressure on the measurement of pelvic floor muscle strength. Clin Biomech (Bristol, Avon). 2014;29(10):1146-50.

2. Bø K, Finckenhagen HB. Vaginal palpation of pelvic floor muscle strength: inter-test reproducibility and comparison between palpation and vaginal squeeze pressure. Acta Obstet Gynecol Scand. 2001;80(10):883-7.

3. Devreese A, Staes F, De Weerdt W, Feys H, Van Assche A, Penninckx F, et al. Clinical evaluation of pelvic floor muscle function in continent and incontinent women. Neurourol Urodyn. 2004;23(3):190-7.

4. Ferreira CH, Barbosa PB, de Oliveira Souza F, Antônio FI, Franco MM, Bø K. Inter-rater reliability study of the modified Oxford Grading Scale and the Peritron manometer. Physiotherapy. 2011;97(2):132-8.

5. Navarro Brazález B, Torres Lacomba M, de la Villa P, Sánchez Sánchez B, Prieto Gómez V, Asúnsolo Del Barco Á, et al. The evaluation of pelvic floor muscle strength in women with pelvic floor dysfunction: a reliability and correlation study. Neurourol Urodyn. 2018;37(1):269-77.

6. Dougherty M, Abrams R, McKey PL. An instrument to assess the dynamic characteristics of the circumvaginal musculature. Nurs Res. 1986;35(4):202-6.

7. Hung HC, Chih SY, Lin HH, Tsauo JY. Exercise adherence to pelvic floor muscle strengthening is not a significant predictor of symptom reduction for women with urinary incontinence. Arch Phys Med Rehabil. 2012;93(10):1795-800.

8. Nystrom E, Antonio FI, Brito LGO, Ferreira CHJ, Nystrom E. Treatment of stress urinary incontinence with a mobile app: factors associated with success. Physiother Theory Pract. 2018;29(9):1325-33.

9. Dinc A, Kizilkaya Beji N, Yalcin O. Effect of pelvic floor muscle exercises in the treatment of urinary incontinence during pregnancy and the postpartum period. Int Urogynecol J Pelvic Floor Dysfunct. 2009;20(10):1223-31.

10. Dougherty M, Bishop K, Mooney R, Gimotty P, Williams B. Graded pelvic muscle exercise. Effect on stress urinary incontinence. J Reprod Med. 1993;38(9):684-91.

11. Sun Z-J, Zhu L, Liang M-L, Xu T, Lang J-H. Comparison of outcomes between postpartum and non-postpartum women with stress urinary incontinence treated with conservative therapy: a prospective cohort study. Neurourol Urodyn. 2018;37:1426-33.

12. Bø K. Pelvic floor muscle strength and response to pelvic floor muscle training for stress urinary incontinence. Neurourol Urodyn. 2003;22(7):654-8.

13. Theofrastous JP, Wyman JF, Bump RC, McClish DK, Elser DM, Bland DR, et al. Effects of pelvic floor muscle training on strength and predictors of response in the treatment of urinary incontinence. Neurourol Urodyn. 2002;21(5):486-90.

14. Burns PA, Pranikoff K, Nochajski TH, Hadley EC, Levy KJ, Ory MG. A comparison of effectiveness of biofeedback and pelvic muscle exercise treatment of stress incontinence in older community-dwelling women. J Gerontol. 1993;48(4):M167-M74.

15. Hahn I, Milsom I, Fall M, Ekelund P. Long-term results of pelvic floor training in female stress urinary incontinence. Br J Urol. 1993;72:421-7.

16. Kim H, Suzuki T, Yoshida Y, Yoshida H. Effectiveness of multidimensional exercises for the treatment of stress urinary incontinence in elderly community-dwelling Japanese women: a randomized, controlled, crossover trial. J Am Geriatr Soc. 2007;55(12):1932-9.

17. Segal S, Morse A, Sangal P, Hirsch N, Kohli N. Efficacy of FemiScan pelvic floor therapy for the treatment of urinary incontinence. Female Pelvic Med Reconstr Surg. 2016;22(6):433-7.

18. Cammu H, Van Nylen M, Amy JJ. A 10-year follow-up after Kegel pelvic floor muscle exercises for genuine stress incontinence. BJU Int. 2000;85(6):655-8.

19. Junginger B, Seibt E, Baessler K. Bladder-neck effective, integrative pelvic floor rehabilitation program: follow-up investigation. Eur J Obstet Gynecol Reprod Biol. 2014;174:150-3.
